# See Change: Overcoming Anti-Black Racism in Health Systems

**DOI:** 10.3389/fpubh.2022.895684

**Published:** 2022-06-02

**Authors:** Adedoyin Eisape, André Nogueira

**Affiliations:** ^1^Department of Global Health and Population, Harvard T.H. Chan School of Public Health, Boston, MA, United States; ^2^Design Laboratory, Harvard T.H. Chan School of Public Health, Boston, MA, United States

**Keywords:** COVID-19, anti-Black racism, design, health systems, inequity, pandemics

## Abstract

Anti-Black racism embedded in contemporary health systems harms Black and Indigenous People of Color (BIPoC) in concert with various diseases. Seemingly unrelated at first, the COVID-19 pandemic is a recent example that reveals how the combined manifestations of anti-Black racism in disease governance, course, and burden exacerbate the historic and still present subjugation of Black people. Thus, such conditions highlight a biosocial network that intricately propagates and consolidates systems of oppression since the birth of the United States of America. In this article, we show how anti-Black racism in conjunction with past and ongoing epidemics exemplify intertwined conditions embodying and perpetuating racial inequities in the North American country. Through schematic visualizations and techniques of progressive disclosure, we situate disease governance, course, and burden as action spaces within a design model that alternates views of organizational strategies, operations, offerings, and people's experiences, supporting an action-oriented discussion in each of these spaces. We utilize insights from this analysis to recommend that public health moves forward, considering more holistic, solution-oriented questions that embrace systemic complexity and people-centered perspectives when seeking to improve health outcomes for all.

## Introduction

A novel coronavirus (SARS-CoV-2) swept the world in 2020, triggering a global pandemic of an acute respiratory syndrome (COVID-19) in humans. In the ongoing wake of this virus, an estimated 5.89 million people have been recorded as dead worldwide as of February 22, 2022, though the actual death toll is much higher due to discrepancies in the documentation (1). In the US, home to ~15% of the global fatalities (1), Black and Indigenous People of Color (BIPoC) populations have the highest rate of COVID-19 infection, hospitalization, and mortality (2). Though this disease was touted as a great equalizer due to the indiscriminate nature of virus transmission, there is a well-documented history of structural racism and violence in the US evidencing how embedded anti-Black bias in existing systems shapes disease governance, course, and burden in communities of color and further exacerbates inequities in health outcomes. While not inherently or biologically more susceptible to disease, the disproportionate rate of poor COVID-19 outcomes among BIPoC communities nationally is a trend that has generationally become commonplace (3).

Seemingly unrelated at first, the COVID-19 pandemic is a contemporary example that reveals how the combined manifestations of anti-Black racism in disease governance, course, and burden exacerbate the historic and still present subjugation of Black people, suggesting a biosocial network that intricately propagates and consolidates systems of oppression since the birth of the US (3). These systems are a byproduct of the institution of American slavery, which was designed to be a permanent, hierarchical organization that utilizes race to ascribe value to bodies in a capitalist production-consumption model. This model enables the production of offerings, including health systems and health-related goods and services, in response to a “free market,” within which governments have little involvement, and people manipulating and/or owning capital goods have greater influence in determining what should be made, why it will create value, how it should be produced, and for whom (4). To guarantee supremacy over other races in this model, white individuals established systems of oppression that gave them exclusive access to and control over various types of capital (e.g., human, social, cultural, political, financial, natural, manufactured, and, more recently, digital), further concentrating wealth and power within the US context through the concept of whiteness.

A critical element that justified practices of oppression was the development and maintenance of narratives surrounding the social construct of racial difference provided the undergirding of white supremacist subjugation of BIPoC populations (5). Encompassed by the various manifestations and mutations of anti-Black racism, this subjugation became the foundation of western expansion (6). To preserve whiteness as a system of power, anti-Black racism was instrumental because it integrated, and continues to integrate, actions of physical and mental violence into every aspect of daily life. When the practice of slavery became a human rights violation in 1808 and then “prohibited” in the United States of America (US) in 1865, the subjugation of BIPoC communities evolved to maintain power structures based on whiteness through legally sanctioned channels (5).

From a public health perspective, Anti-Black racism can be considered a societal disease that synergistically oppresses and marginalizes Black populations, compromising their health in conjunction with various conditions present within and across health systems. This is not new information. In fact, the complex health implications of racism have been documented and disseminated for over 200 years (7). But, without proper recognition and analysis of how structural racism manifests as an underlying condition in health systems, current response paradigms will continue to perpetuate inequities that foster synergistic deleterious conditions (8). As Marie Chisholm-Burns's son asks, “If people are all connected, and racism over the years in the United States has claimed the lives of many, just like COVID-19 has, why are we not racing to find a cure for racism with the same vigor that we are racing to find a cure for COVID-19?” [(9): p. 1537].

Lack of action is not caused by a lack of knowledge. To act differently, individuals and organizations must see differently.

In the last decades, health-related organizations have substantially used design knowledge when conventional approaches developed by medical science and public health further expand gaps between research and practice rather than supporting practical actions. Initially as an outsourced capability, these organizations are increasingly trying to internalize design know-how predominantly in the implementation stages to improve the effectiveness of service delivery.

Nevertheless, organizations from across industries have used design competencies to observe and directly interact with people in their daily life to increase empathy and gain a better understanding of their aspirations and related problems; use participatory approaches to co-create early-stage prototypes that help participants discover new opportunities for value creation across all stakeholders involved in their processes; apply abductive reasoning, complementary to deductive and inductive, that ensures exploration of the future rather than benchmarking the past; and visualize ideas in the forms of sketches and diagrams to make abstract concepts easier for all participants to understand different viewpoints; among other things that enable organizations to frame problems and expand options for making people's lives better (10, 11).

While there is a growing interest from agents in the health industry to better understand how to leverage design to promote more systemic, population-level change, the goal of this article is not to prove the value of design to health-related organizations dealing with complex social challenges. Nor is it to prove racism as a social structure conditioning public health outcomes. Instead, we work at the intersection of public health and design, leveraging the formal analytical structure of the former with the systemic yet pragmatic, solution-oriented approach of the latter to conceptualize how combined manifestations of racism continuously worsen health outcomes for BIPoC in the US.

Through schematic visualizations and techniques of progressive disclosure, we situate disease governance, course, and burden as action spaces within the structure of the Whole View (10), a design model that integrates viewpoints of organizational strategies, operations, offerings, and people's aspirations and related problems, and supports an action-oriented discussion in each of these spaces. We utilize insights from this analysis to recommend that the field of public health moves forward, considering more holistic, solution-oriented questions that embrace systemic complexity with people-centered perspectives when seeking to improve health outcomes for all. In doing so, organizations might be better prepared to reveal the root causes of structural issues and present opportunities to create transformational change toward more equitable health outcomes that have not previously been considered. Although we focus on the experiences of Black populations, we believe these arguments can be contextualized beyond this demographic to encapsulate similar experiences in Indigenous and other communities of color, and/or different stances of marginalization.

### The Role of Anti-Black Racism in the Standardization of Health Systems

**Figure 1:**
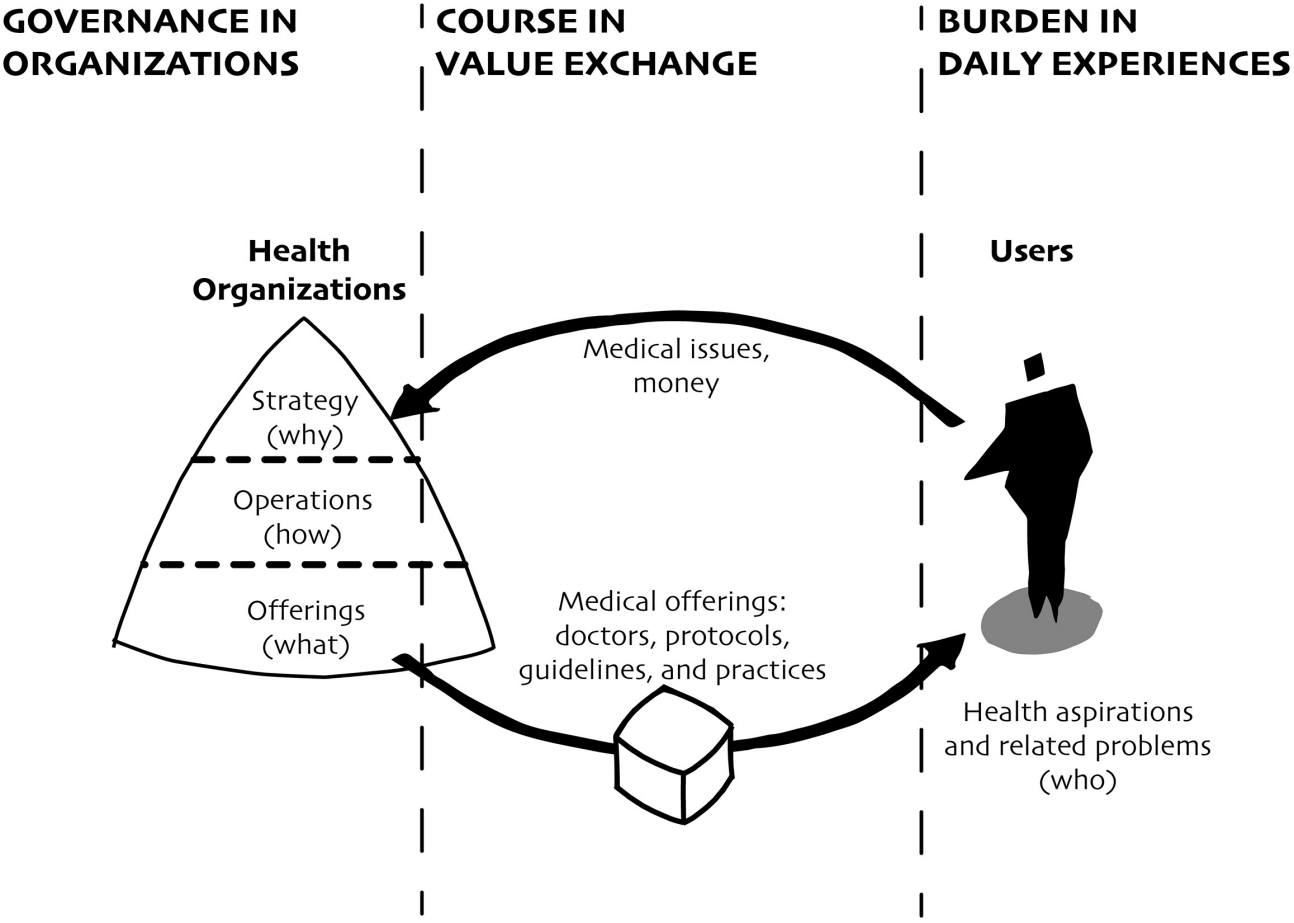
Health-related organizations became the primary agents governing diseases, influencing their courses through medical offerings that respond to specific medical issues and condition how individuals experience their burden. In this diagram, health-related organizations are represented by a triangle divided by strategies (why), operations (how), and offerings (what). The exchange of value is represented by the arrows and related labels. Individuals (who) are represented by a human figure.

Mass production, mass markets, and mass media have been the underlying forces enabling health-related organizations to become the primary agents governing health systems in the US. While different disciplines have created several frameworks to describe an organization, four fundamental questions and related areas have informed their contributions: what to make? (offerings), who is it for? (users), why will it create value? (strategy), and how to make it? (operations). To achieve their purpose, health-related organizations make health systems through various combinations of people, objects, environments, messages, services, platforms, and infrastructures. In turn, these systems are used by the same organizations to strategize around opportunities for value creation (why), efficiently operationalize their activities and effectively fulfill their purpose (how) to meet specific health needs of targeted populations (who) (12). In order to grow and scale impact, health-related organizations have generated standardized medical protocols, guidelines, and practices (what) that were predominantly informed by scientific knowledge on physiology, chemistry, and specific behaviors that directly influence a person's health (Figure 1). Developed by and operationalized for white experts researching and responding to health issues presented by experiences and ideologies of white populations, these standards were adopted countrywide and continue to govern the health systems. For example, Puzan shows how “nursing participates in, reproduces, and resists the detrimental practices associated with white cultural privilege” and shares “some instances of its personal and social costs” (13).

While significant advancements in these standards have improved public health, contemporary health organizations increasingly face friction between behavior change expected by science-based protocols, guidelines, and practices and the plurality of how the people living in the US experience life. This friction is especially evident in critical conditions faced by Black populations. The entrenched anti-Black racism in health-related organizations prevents them from incorporating considerations about how past and current injustices condition disparity in future health outcomes, especially in times of crisis.

For over centuries, health-related organizations have used anti-Black racism as a tool to advance white supremacy and uplift those who benefit from it. The many manifestations of this tool contribute to a continuous onslaught of offenses to the human dignity of Black people, both intentionally and unintentionally. The malleability of these manifestations and how they change to bolster this system work to negate legitimacy claims and demonstrates the extent of this problem. As we have seen with this pandemic, there are many situations when the daily experiences of Black populations directly influence their health outcomes but are not captured by science-based protocols and regimens present in existing health systems.

Following, we draw on historical and ongoing contexts to reveal patterns of racist manifestations influencing the three action spaces: disease governance through the strategies, operations, and offerings of health-related organizations, disease course through standardized exchange models between these organizations and the individuals they intend to serve, and disease burden through lack of knowledge about people's aspirations and related problems. Through progressive disclosure, we expand upon the model presented in Figure 1 to situate how combined these three action spaces catalyze the inequitable outcomes endured by Black populations in the US.

## Disease Governance

Health governance lies at the center of efforts to quell the destruction caused by deadly diseases, including those causing pandemics. Dimensions of health governance relate to a broad spectrum of steering functions carried out by individuals in authority positions within health-related organizations making decisions to safeguard population health (14). Examples of governing functions include partnerships, regulation, transparency (strategy); accountability, participation, and consensus (operations); generating information, formulating policy, and systems design (offerings).

In seeking to perform health governing functions, decision-makers tend to be outcome-driven, benchmarking the past rather than process-oriented, seeking alternative futures that can improve health systems for all (15–17). Consequently, the adverse outcomes of choices made by governing agents reverberate more heavily in communities whose realities differ from those that informed the design of health-related organizations and their standardized systems of offerings. Such dependency on “what has worked for specific populations” prevents governing agents from adopting a more holistic lens and realizing “what must work for all.”

In turn, countless instances of inequities within medical delivery must be explored as a product of choices that uphold systemic anti-Black racism. Such framing grounds agency and accountability to individuals in a position of authority making decisions within health-related organizations. While the ‘who’ can often seem amorphous, existing health systems have been designed with a clear hierarchy between national, state, and local leadership that dictate or predicate help upon reliance on white supremacy (18). In fact, an analysis of the various etiological pathways involved in health governance demonstrates a clear choice architecture embedded in organizational strategies, operations, and offerings that contribute to the subjugation of Black populations over generations (19). Understanding these various levels and spheres of influence provides a foundation for uncovering and intervening in how power, especially within or withheld from marginalized communities, manifests in health and illness and intrinsically consolidates and propagates a system of subjugation and oppression (8).

**Figure 2:**
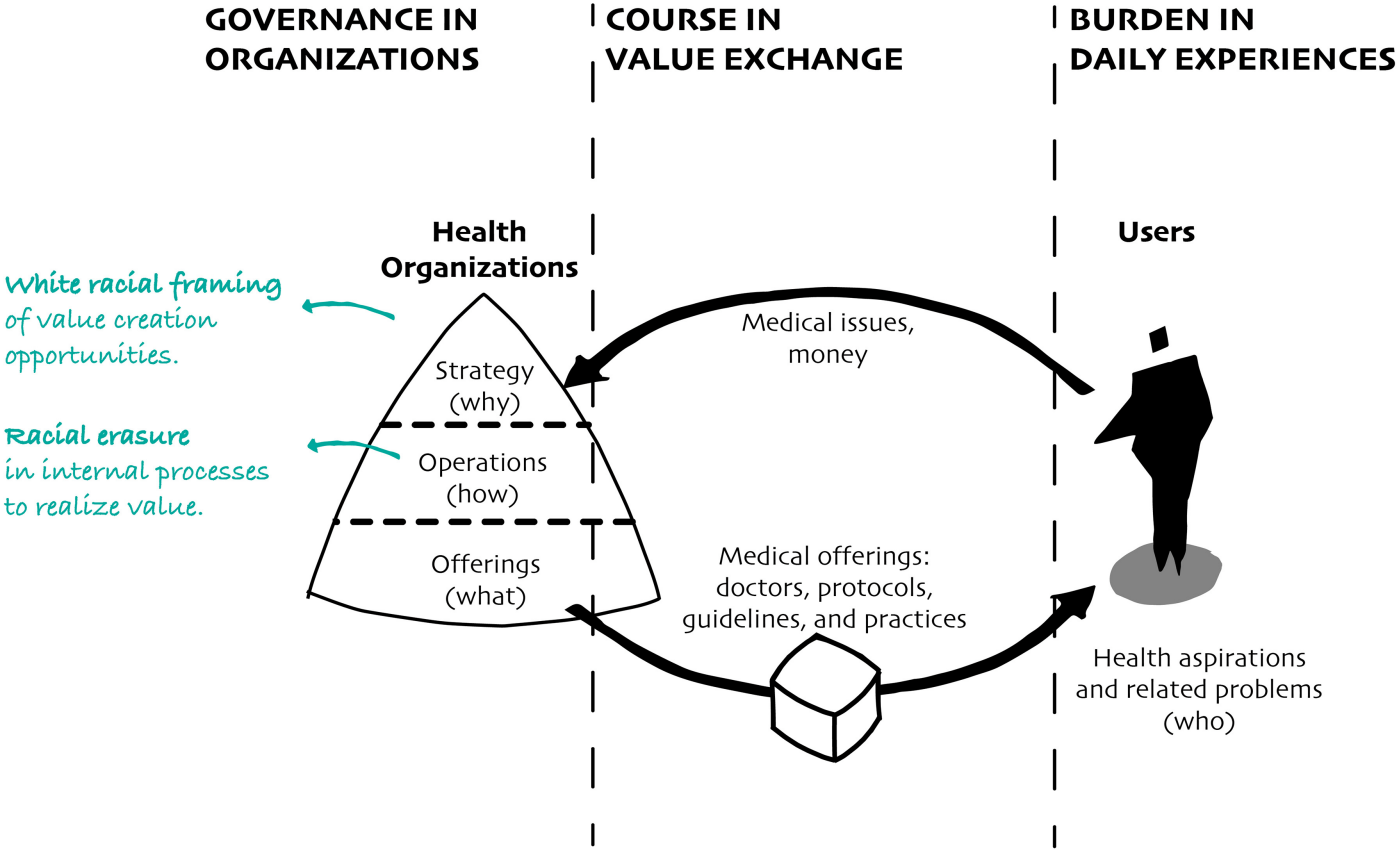
Primarily designed and led by white individuals, health organizations create strategies and operations that respond to why and how they envision value being created to combat diseases.

Health governance is crucial in any disease response. However, in times of crisis, the enduring toleration of anti-Black racism that underpins values, beliefs, attitudes, norms, structures, and socio-political contexts becomes more explicit in decision-making frameworks that help determine health outcomes. Below we outline two health governance properties that prevent decision-makers in US health-related organizations from promoting transformational change: a strategic “white racial framing” and an enduring “racial erasure” in organizational operations (Figure 2).

### White Racial Framing

The dominant value system in the US “operates to normalize the white experience, frequently excluding all other racial groups” (20). A strategic ‘white racial frame’ in overarching ideologies, images, stereotypes, assumptions, and narratives within health-related organizations reinforces a value system that elevates opportunities to create value for white populations while underpinning trauma for non-white populations (21). Such biases have been preserved and reproduced to further obscure an understanding of the relationship between choice and the health effect of structural racism (20). While the current moment of civil unrest has yielded numerous statements denouncing racism and committing to progress on equity and racial justice, which partially represent the enduring fight for improving outcomes for all, very few endeavors critically assess harmful structures and the role of power in the sustainability of such efforts (22).

For example, many educational and research institutions within health-related industries commit to greater racial diversity during recruiting processes (23). In the past decade, many have considered adopting internal racial and gender-related Key Performance Indicators (KPIs) as a positive strategy for increasing diversity through new hiring processes (24). However, without proper representation and advocacy to actively support marginalized or politicized groups, not as a group member but in solidarity with their struggle, through tangible mechanisms for power redistribution, these efforts focus on the same body of proven players, overlooking emerging scholars of color while simultaneously obstructing opportunities to effect change (25, 26). The subjective process of talent searches within educational institutions “typically mirror the networks of those leading the search,” demonstrating a “self-referential decision-making process[es]” contributing to the large number of racial minorities that are left out of these procedures (27). As a result, the overall number of underrepresented people in positions of power remains unchanged as the same people are recycled throughout the existing recruiting system (20). With a white racial framing as the norm, recruiting processes within educational institutions restrict the notion of success to weed out those who push against that norm, thus resisting and undermining avenues for new approaches to improve health outcomes for all (28).

Another strategic condition involves recognizing and supporting scholars based on the number of citations of their work and contributions to specific journals. Within this value system, emerging scholars are encouraged to build on existing work deemed successful rather than expand existing practices to embrace other forms of impact that do not gravitate around norms of whiteness. But what happens to scholars interested in conducting research around anti-Black racism when less than 1% of articles published in the top 4 medical journals over the last 30 years included the word “racism” anywhere in the text? (29). How can scholars connecting racism to health outcomes advance their work and impact?

Indeed, a history of protests and unrest has “welcomed” Black individuals into positions of power, warranted costly initiatives, and uncomfortable conversations related to equity. These initiatives have garnered minimal progress toward restructuring structurally racist and inequitable health systems in the US upheld through various institutions (27). Still, in the face of unyielding institutional exclusion and power inequalities, many equity efforts manifest as superficial, prompting “tokenist” views of critical equity, inclusion, and belonging efforts and/or perfunctory analysis of racism to appease surface calls for change.

### Racial Erasure

To support a strategic white racial framing, leaders within health-related organizations foster racism-evasive rhetoric in their operations. Racial erasure is a condition that rejects the explicit naming of racism and, in doing so, dismisses attempts at addressing its realities. This rhetoric is centered on how external racial ideologies of “color-blindness” interact with internal organizational culture to produce racism-evasive responses that claim neutrality (30). Such a perspective has perpetuated the ‘color-blind’ trope that skin color should not matter in discussing complex challenges and issues in disease governance. By adopting a reductionist understanding “that racism is limited to individual intentional acts committed by unkind people” (31), decision-makers infuse a defense mechanism against practices of racial oppression and injustice with the belief that racism is fundamentally an issue of the past, and those who continually call out or fight against it are troublemakers “playing the race card” (30). DiAngelo has expanded this condition from the perspective of “white fragility,” or the disbelief and defensiveness that white people display when their ideas about race and racism are challenged as well as when they feel guilt for or implicated in white supremacy (32). The author suggests this dynamic promotes behaviors such as argumentation, silencing, and withdrawal from participation in terms of racial justice. As a result, when Black people voice the discrimination they experience, including racist bullying, harassment, trauma, and how racial hostility informs connection to place, they are often silenced or denied by organizational leaders and/or colleagues (21).

Still, the impediments of racial erasure go beyond racist practices within resistant operational processes. Progressive agendas of inclusivity can also reaffirm the racism-evasive rhetoric in divisive acts. The perceived “need to downplay talk on racism for the sake of solidarity” serves as an example (30). Individuals in positions of authority have the power to determine when, where, and how Black people are allowed to reflect, share on, and defend race-based topics without acknowledging that status and privilege (21). This condition is based on classism and elitism, wherein the strong, often unconscious, white preference for Black mediocrity demonstrates how far-reaching anti-Blackness manifests in health-related organizations shaping public health agendas (33). In turn, well-intentioned initiatives contribute to a vicious cycle of resentment and speculation of a “lowered bar” for underrepresented professionals of color, even when they are otherwise overlooked” (27). These are examples of how various modes of racial erasure have ingrained racial illiteracy in health-related organizations, promoting an ahistorical, asymmetrical, and amoral interpretation of drivers and manifestations of racism that condition health outcomes in Black populations (34).

### Advancing Governance Through Action

The strategic and operational manifestations of racism described above are examples of structural barriers to pursuing more equitable health outcomes for all. Both white framing and racial erasure are robust forces of racism contributing to transferring bias into new offerings, including medical professionals, products, environments, messages, and services, which form systems that perpetuate violence and marginalization of Black populations. Without addressing the mechanisms that replicate systems of power that exclude or obfuscate Black people and the lived experience of structural racism, calls for racial justice and equity will continue to fall on inherently complacent ears. When addressing the insidious nature of structural violence, it is fundamental to transition from the rhetorical to the actionable. Individuals in positions of authority must commit to exposing and rectifying mechanisms that perpetuate the violence and trauma of structural racism in manners that promote accountability. To this end, a commitment to stop reinforcing racist power structures must involve intentional power redistribution to effectively imbue an actionable racial justice and equity agenda within health governance.

## Disease Course

Disease course relates to the pathways a disease takes that determine how it behaves in an individual or population over time. Understanding disease courses offers an essential mechanism for limiting the life-threatening nature of a global health crisis, such as a pandemic. Evolving analysis of COVID-19, for instance, demonstrates the level of inquiry and urgency required to deter the most severe population outcomes (35).

**Figure 3:**
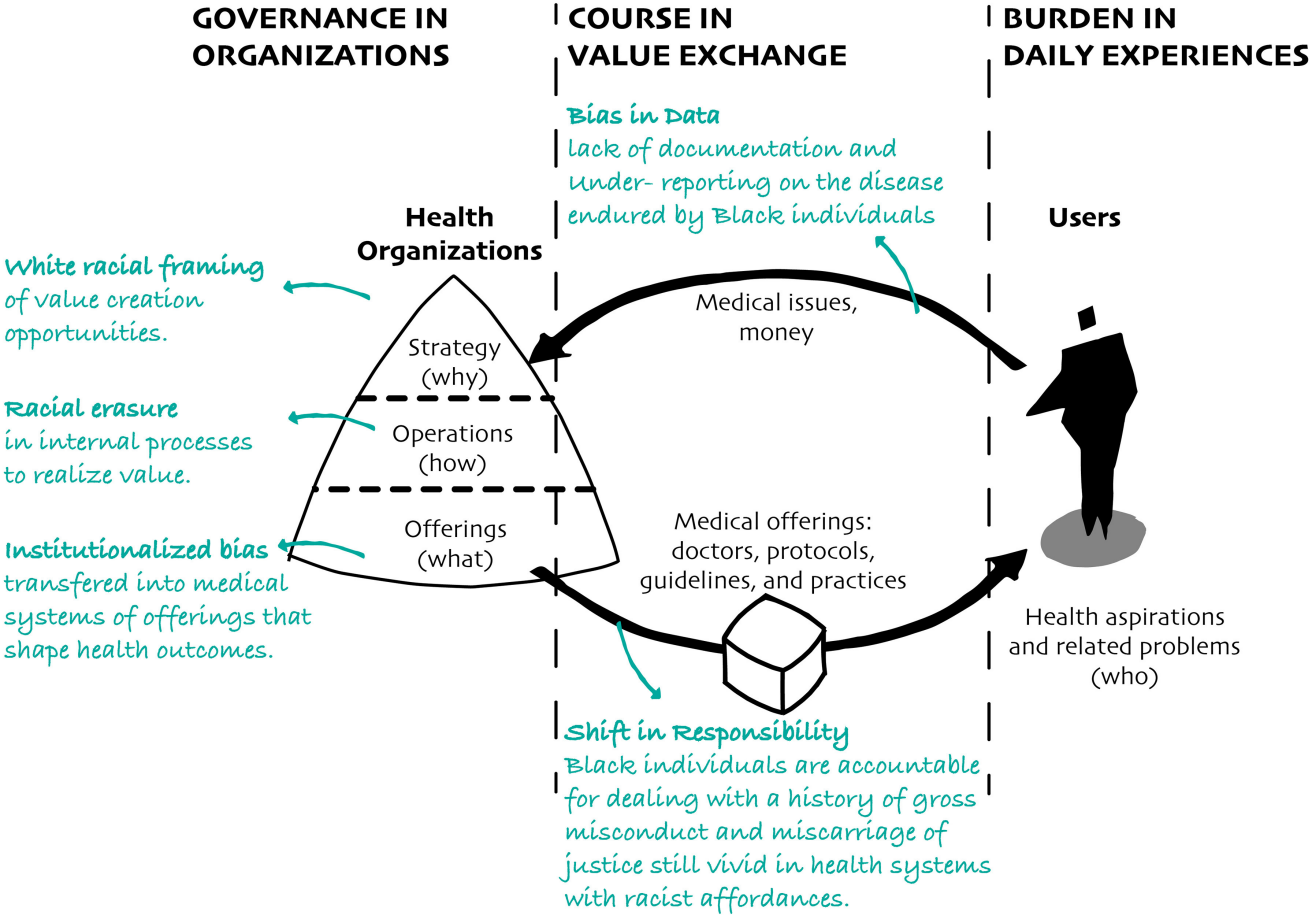
Health organizations create systems of offerings based on the information available to them. Because they lack proper documentation on diseases endured by Black populations, they transfer racial bias into medical offerings that shape the experiences and health outcomes of Black populations.

In contemporary health systems, the course of a disease is conditioned by how health-related organizations capture and create value, understand the medical issues caused by that disease, and make systems of offerings useful to help individuals who are able to afford such offerings overcome related threats, respectively (36). In this value exchange between people sharing their perceived problems and organizations trying to create solutions, anti-Black racism becomes a powerful force driving the various trajectories of a disease. The enduring racialization, historicization, and mechanization in the processes shaping this value exchange undermines the ability of health-related organizations to improve the health of all (37). In fact, when health-related organizations lack proper consideration of or, worse, ignore systemic and historical conditions that concentrate risk in Black populations, they create systems of offerings that accelerate the spread of that disease through sanctioned channels and standardized mechanisms (34, 38). In doing so, disease course becomes an issue of race, centered on individuals, rather than racism, centered on organizations and key stakeholders. The erasure of Black populations in data and focus on Black responsibility in health systems are two significant dimensions influencing how health-related organizations perceive the manifestations and implications caused by a disease in an individual or population, and respond accordingly (Figure 3).

### Bias in Data

The presiding reliance on existing medical evidence and interventions deemed successful suggests that available data plays a fundamental role in determining future interventions to improve health outcomes. But how should health-related organizations design equitable health systems when there is a limited account of the disparate experiences of disease in Black populations in the US?

For example, the under-reporting and lack of documentation on the disease course endured by African Americans impedes an account of the actual incidence of morbidity and mortality resulting from the 1793 yellow fever epidemic in Philadelphia (39). Likewise, there is a “shockingly sparse” record of the effect of the 1918 influenza pandemic on Black individuals (40). While significant progress has been made in technological advancements and computational mechanisms for data collection and analysis since these two crises, the incompleteness of racial and ethnic data during the ongoing COVID-19 outbreak evidences how health-related organizations continue to inadequately capture disease course, including misunderstanding of medical failings faced by Black populations, lack of services and accessibility to affordable health care. Consequently, they neglect to help mitigate inequities promoted by current health systems (41).

Even when these agents accurately account for cases and deaths of Black populations concerning widespread diseases, the segregation of counts from the dynamics of daily life leaves data isolated from meaningful application to improve health outcomes. As metrics of population-level intervention success primarily rely on aggregated data, this process can obscure a wide range of factors influencing the perception of problems, actions, and outcomes.

In addition to building upon an existing body of work, current data collection approaches exercise asymmetric control over what questions are asked and what metrics are measured. Given the current state of disease governance, what information is deemed worthy of collection, who guides inquiry and can access it, how it is acquired, and how stories are told, all carry racial biases ingrained in health systems' response (42) and perpetuate power inequities (43). Without data that can holistically account for and respond to manifestations of anti-Black racism, health-related organizations will continue to fall short in their understanding of how and why certain diseases disproportionately affect Black populations.

A population's (in)visibility in data indicates social standing, and those with more power within society weigh more heavily in data capture and documentation related to public health that further inform organizational offerings in response. Thus, under-representation in data collection and documentation efforts invariably impact problem-framing and solution-finding processes that affect health outcomes for all. This reinforcing loop suggests that health-related organizations cannot adequately incorporate emerging, dynamic conditions for those most affected by their decisions and may fail to elucidate what is effective for Black populations, consequently contributing to widening inequities in disease courses.

### Shift in Responsibility

Deemed as “outliers” and absent in the data collection process, Black people with experiences that differ from conventional white frames are often overlooked, under quantified, and invalidated in the design of medical protocols, guidelines, practices, and other organizational offerings that shape disease courses. The resulting lack of trust in health-related organizations and the health systems they create has been identified as a critical issue in determining disease courses. Rather than having their experiences acknowledged and used as the basis for designing better interventions for all, Black individuals have been continuously held accountable for educating and advocating for themselves amidst the fiction that contemporary health agents “do no harm,” regardless of their past experiences.

Today, there is an extensive history of gross misconduct and inequity transferred into medical offerings that evidence how health-related fields are complicit in providing inferior care to Black people and continue to exploit and degrade communities of color and other marginalized groups (44, 45). For example, recent studies concluded that Non-Hispanic blacks with dementia had approximately double the risk of underdiagnosis then their non-Hispanic whites counterparts (46), and that chances of dying due to pregnancy-related causes are still two to three times higher for Black, Native American, and Alaskan Native women than white women (47). Many other documented examples can be found, such as the legacy of Anarcha, Betsy, and Lucy, who should be known as the true Mothers of Gynecology, or in racial distinctions in drug therapy taught at the university level that perpetuates inequality in treatment (45).

At the core of these issues is a deep-rooted disparate system of offerings that fails to care for Black individuals. For many Black people, almost all medical interactions involve, in some way, shape, or form, a battle to self-advocate or fight for baseline, adequate care. Even when Black individuals explicitly state and use their experiences, they often must shoulder the burden of proof as the requirement for data, which provides a pivotal mechanism to bolster whiteness as the norm. The paradoxical condition involving the reliance on available data and lack of data represents purposeful barriers placed to exclude the experiences of Black individuals from the record and a direct manifestation of racism in health-related organizations. The death of Dr. Susan Moore, a Black physician whose battle against COVID-19 was impeded by a deeply ingrained racial bias and discrimination within the medical field, provides a specific contemporary manifestation (48). As seen with many Black populations during their interactions with western health systems, medical professionals minimized her pain and withheld treatment. Her educational and professional status was not enough to protect her from a system of subjugation that devalued her pursuit of health and quality treatment as she pleaded for her life.

The situation faced by Dr. Susan Moore should not be considered as an example for building on an existing yet devalued body of work concerning racism in health systems. She was a person, and hers is a story of a woman who understood medical science as well as her symptoms but was ignored and lost her life due to a failing, racist health system. Similar to Amy Bach's assessment of state criminal courts, when health systems regularly permit basic process failures through their offerings, medical professionals become more accustomed to patterns of lapses of injustice and can no longer see their role within them (49). As a result, the miscarriages of justice that come to light (as previously documented in the Tuskegee Syphilis Study or forced sterilizations of Black women) tend to focus on single-player blame rather than systemic critique that exposes how health organizations continue to hold Black individuals accountable for solving structural issues related to racism embedded in contemporary health systems.

This is critical to health-related fields because it challenges the nature of evidence informing the design of medical offerings, including trained doctors, hospital environments and clinics, healthcare products and services, and how racial bias in medicine also lies in the most covert mechanisms. For instance, how can Black people trust health systems in the current pandemic when studies have shown that pulse oximeters, a standard machine that measures blood oxygen saturation levels in hospitals worldwide, regularly have inaccurate readings on darker skin, contributing to differential outcomes in Black and white patients? (50).

The cases presented in this section illustrate how racist affordances, or properties of an offering that give its users the possibility to take an action, regardless of their ability to perceive this possibility (51), are embedded in current health systems. They determine disease course and cannot be eased by superficial efforts, such as information campaigns focusing on transparency and relying on the work of leaders of color to solve vaccine hesitancy. Instead, inadequate and shallow attempts force the onus of changing health system on marginalized and oppressed communities without fully addressing the conditions currently reproducing distrust in health care. They also demonstrate that communities of color have not been given sufficient reason to redevelop trust in the medical industry, given marked differences in present-day health outcomes for these communities. Thus, new services and communication strategies designed to increase vaccine buy-in in Black populations demonstrate the ineffectiveness of current systems in creating procedures and policies that oppose the marginalization of Black communities. As long as diverse modes of illness prevention and medical decision-making continue to be based on a standardized notion of disease course defined by whiteness, people of color will be overlooked in—and further marginalized by—the service offering landscape and public health will continue to be unprepared to fight the next crisis affecting the health of all populations.

### Reorienting Disease Course

Data is not neutral, and data in a vacuum is not enough to quell the flow of this structural violence experienced by communities of color. To advance on an equity agenda without accounting for the under-representation of Black experiences in data streams, both in seeking and receiving care, means allocating resources in interventions that are not representative of and might undermine transformative anti-racist practices. If confrontations toward anti-Black racism remain focused on the individual over organizational responsibility, unceasing failures in health systems will continue to guide disease courses in marginalized communities.

What if health-related organizations critiqued power and positionality routine in data collection methods and accountability measures? Would this provide an opportunity to shift goal setting toward more equitable practices? As stated in the Chicago Beyond report, “Why am I always being researched?”, “If we want to avoid perpetuating the same systemic problems and structural biases that have persisted in communities of color for generations, and which Covid-19 is now presenting to us in full color, we need to change our orientation in how we produce, analyze, use, and assess the validity of evidence.” [(42): p. 2]. A critical step in this process is to expand an understanding of the lived experiences of Black populations and how racist organizations and their systems of offerings worsen outcomes in these communities.

## Disease Burden

Disease burden is a familiar concept used in public health to examine the extent to which diseases, injuries, and risk factors contribute to loss of health and death throughout a person's or a population's lifespan (52). It demonstrates an important indicator of severity, endurance, and overall impact of a pandemic on people. Many health-related indicators are used to measure this phenomenon, including potential years of life lost due to premature death, equivalent years of “healthy” life lost by being in states of poor health or disability, and loss of quality of life caused by diseases and disabilities. Most health-related organizations leverage these and other supporting indicators to determine the cost-effectiveness of existing health systems and explore interventions for expanding disease treatment capacity programs (53). Nevertheless, there are many situations when their daily activities and behavior directly influence health outcomes but are not captured by health-based protocols, indicators, and regimens present in existing health systems.

People act by fitting information, resources, and other offerings available to them to create daily life experiences they understand and help achieve their aspirations in meaningful ways. Without capturing critical information about other contextual elements that condition everyday experiences and provide the context for the manifestations of disease burden, health-related organizations can become competent in making incremental changes to current health offerings, but will likely fall short in supporting the transformations required to improve the larger populations' quality of life.

**Figure 4:**
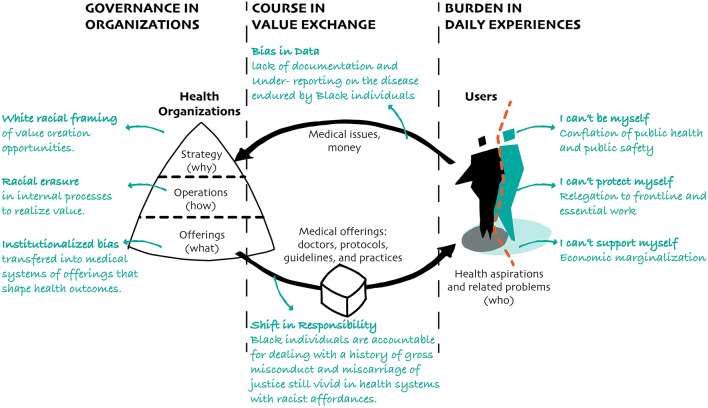
The diagram above illustrates how critical aspirations of Black individuals, including those related to their freedom, safety, and economic security, become conditioned by health systems that carry racist affordances in disease governance and courses.

For marginalized communities deemed as fodder for the system, years of “healthy” life as well as the quality of life lost as a product of this marginalization can contextualize the shifted weight of disease burden for certain populations. Countless examples of how lived experiences conditioned by racial inequities compound disease burden illustrate the deep-rooted power inequities that force Black communities into high-risk health conditions over generations. Below we highlight three patterns of experiences and related conditions, using past and ongoing pandemics as examples; (A) I can't be myself: conflation of public health and public safety, (B) I can't protect myself: relegation to frontline work, and (C) I can't support myself: economic marginalization (Figure 4).

### I Can't Be Myself: Conflation of Public Health and Public Safety

When the outcomes of Black populations are posited as divergent norms, public health practice can be predicated on the policing of people in a manner that is susceptible to further marginalization and takes away their freedom to be themselves. As demonstrated by race-baiting propaganda designed during the 1793 yellow fever epidemic in Philadelphia, Black communities were vilified and disparaged. With a message intentionally framing Black individuals as people taking advantage of the distressed pandemic conditions (54), this propaganda further contributed to fear of a “Black threat” and fostered a racialized perception of public safety. Likewise, uncertainty about disease transmission during the 1918 influenza pandemic manifested in eugenics-based arguments to justify racial hatred and reinforce segregationist subjugation strategies (55). During the current COVID-19 pandemic, racial disparities in community criminalization and legitimization of police brutality heighten community dysregulation and distress (56), especially in Black communities living in marginalized territories. Such disparities made it difficult for many Black families and businesses to leave high-risk conditions readily while simultaneously promoting internal displacement and community dysregulation (57–59). With several supporting structures centering relief on behavior change as a condition of aid and targeting low-income and Black populations as the disease vector, a deficit approach in public health response to the crisis was bolstered (55).

Within a myopic framing of what is socially acceptable and who gets to experience what, public safety has been used as a dog whistle to obscure the visibility of bias in values and operations under the guise of governing health. In addition, racialized arguments for community dysregulation, unequal living conditions, and ill-maintained infrastructure have been a powerful means to obscure systemic faults and maintain mechanisms of segregation (60). If anti-Black racism continues to be channeled through a conflation of public health—as a means to promote positive population-level health outcomes—and public safety—as safeguarding populations from threats—the current and future pandemic responses will inherently sustain multigenerational oppression and depression of health prospects for marginalized communities post-pandemic.

### I Can't Protect Myself: Relegation to Frontline and Essential Work

What began as misinformation surrounding race and innate immunity from disease became an evident reliance on Black communities at the frontline of pathology, voluntarily or otherwise, to control outbreaks without recognition (61). Black communities, tasked with nursing the sick and burying the dead from the white populous during the 1793 yellow fever epidemic, were forced to give care to those who further supported their subjugation as the white medical bourgeoisie refused to treat colored communities (62). The 1918 influenza pandemic encoded this manner of exploitation by the increasing premium of African American medical staff in supporting disease management without support or care in return (41). The current COVID-19 pandemic response reflects a similar scenario as rhetoric shifted from “keeping all Americans safe” to “reopen businesses at all costs” after data demonstrated pathology concentration in communities of color (63). With 80% of Black workers across the United States not able to protect themselves working from home, their essential status engenders their sacrifice as a cornerstone of crisis management without adequate recognition or compensation, i.e., underpayment, lack of health care coverage, disrespect for human rights (33, 64).

Throughout pandemic records, this preference for Black individuals at the forefront of disease demonstrates how underlying racial capitalism, in which strategies for the extraction or accumulation of wealth based on racial hierarchies, concentrate disease burden for Black communities, especially during times of crisis (65). When there is a prominence of marginalized communities in frontline and essential positions, it demonstrates the intentional decision to treat Black individuals as expendable. This practice becomes an institutional reproduction of violence and oppression rather than crisis management. If economic prosperity is placed above Black lives, this racial approach to capitalist health systems of production and consumption will widen health disparities and deepen the impacts of inequities experienced by Black populations.

### I Can't Support Myself: Economic Marginalization

The rapid response landscape is rife with examples of how structures and systems continue to exclude historically underserved populations. The marginalization of Black communities has contributed to stagnated economic growth as well as the perception of low-wealth and lack of internal productive capacity. For more than a century, Black businesses have been subject to discrimination in accessing financial support, such as requirements of extra documentation or assets to secure external financing. For example, only 1% of Black business owners receive loans in their founding year compared to 7% percent of white business owners (66). Even highly-rated businesses in minority areas are perceived as unsafe or undesirable and are less likely to be patronized, putting them at a competitive disadvantage to white counterparts. During the COVID-19 pandemic, these structural roadblocks continue to manifest in the marginalization of Black populations, exemplified by the 90% denial rate of Black and Latinx small businesses for Paycheck Protection Program (PPP) funding (67). Moreover, the 41% of Black-owned small businesses that closed during the pandemic, when compared to only 17% of white-owned small businesses, becomes yet another evidence of how federal relief operates within racist historical frameworks rather than challenging them (68). Thus the exclusion of communities of color from support mechanisms compounds the impact of short-term setbacks with long-term implications.

### Contextualizing Disease Burden

What makes marginalization so unique is that though it manifests in a power system based on whiteness and those who uphold it, the true extent of its deleterious effects is concentrated in communities being marginalized. Thus, the routine violence and trauma experienced by Black populations are compounded by systemic devaluation and continued impediments to personal autonomy, including the added emotional and cognitive effort required to maintain health and well-being on a daily basis (21). The conflation of public health and public safety, frontline worker status, and economic marginalization are three conditions that exemplify how anti-Black racism governs such daily experiences and prevent them from being, protecting, and supporting themselves, respectively. In turn, years of life lived under such disabling conditions create an inter-generational burden that erodes the ability of Black populations in the US. to realize the most basic standard of living afforded to their contemporaries. Without expanding the disease burden to include an understanding of how anti-Black racism shapes the daily experiences of Black populations, public health will continue to fall short in promoting accurate rectification of deep-rooted power inequities that provide context for its interventions and practices.

## Expanding Public Health Through Design

Although racial inequalities conditioning the health of the public have been discussed for centuries, leaders across health-related organizations still debate their progression rate and consequences instead of committing to transformative action. Racism is so ubiquitous that it lives in societal blind spots, yet insufficient action is not caused by a lack of knowledge or great ideas. Instead, it becomes clear that processes largely driven by formulaic science, rigid data schemes, and the stringent analysis of economic, demographic, environmental, and medical information can overlook the lived experience of policy and protocol.

As historian John Henrik Clarke once said, “if slavery is your baseline for understanding Black people, then everything else looks like progress” (69). Though health systems have evolved and advanced, the same cannot be said for the frame of reference of what progress should or could look like for marginalized populations. Today, academic and policy professionals who are believed to have reliable knowledge of the “right” ideas and “best” practices, tend to frame, formalize, standardize, and evaluate the successes of transformative projects that condition people's daily experience. Over the years, though these standards have supported the field's progress, they also contribute to the unintended consequence of creating professional silos and biases that impact public health. Examples include fragmented healthcare delivery (70), barriers to information sharing (71), and the fallacy of “Expert Immunity,” or the incorrect belief that experts are impartial or immune to biases due to their expertise (72).

The conceptual analysis presented in this article suggests that looking at disease in tandem with manifestations of white supremacist systems can challenge a limited understanding of progress by helping surface the root causes of structural inequalities compromising positive health outcomes in Black populations. It does so by providing an alternative decision-making framework to support the institutionalization of a more holistic yet pragmatic progressive rhetoric that can not only improve the experience of Black individuals as end-users but, more importantly, recognize they must be involved in all stages and parts of contemporary health systems. Nevertheless, most large-scale transformational changes, such as those needed to overcome racism in disease governance, course, and burden, can still face significant resistance from individuals currently benefiting from the status quo, further institutionalizing the systematic marginalization and rejection of meaningful work that are not representative of those in positions of power. Without altering the racist beliefs and habits embedded in strategies, operations, and offerings conditioning modern health systems in the US, it is unlikely that emerging racial justice movements and policies will bring about the desired changes.

### To Act Differently, People Must See Differently

Recognizing that today's complex and ambiguous challenges require more than incremental steps to existing structures and cannot be adequately addressed by existing practices, several organizations are adopting design as a complementary approach to move forward in the face of uncertainty. Over the last 25 years, the field has seen the emergence of two unique specialties that have embraced more holistic approaches to problem-solving: “human-centered design” and “strategic design” (10). Human-centered design (HCD) incorporates considerations of end-users, prioritizing the understanding of people's activities and aspirations to ensure solutions are effectively improving their experiences. Strategic design links insights gained from HCD approaches to the volatile world of business and flexible production. Combined, HCD and strategic design led to frameworks and methods that help organizations identify people's values, behavior, and habits shaped by culture and link them to the formal frameworks from science, business, and engineering.

**Figure 5:**
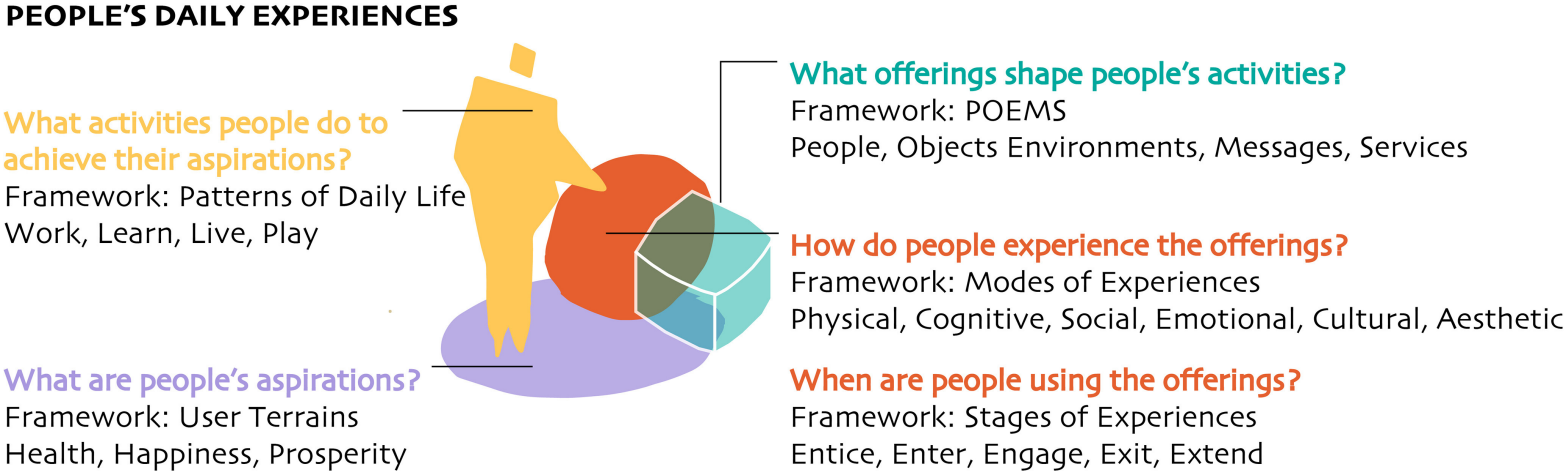
The diagram above shows critical questions that designers ask to understand people's experiences and related frameworks they use to organize information gathered during research. For example, POEMS, an acronym for people, objects, environments, messages, and services that compose systems of offerings, and User Terrains, that helps organizations understand their users in the context of their aspirations, rather than solely socio-economic and demographic standards.

For instance, to understand how people use various components that shape existing systems, design offers two other frameworks considering people's experiences in a range of modes and stages. Modes of Experiences is a framework that involves cognitive, social, cultural, physical, emotional, and aesthetic factors that condition how people interact with specific offerings (73). Stages of Experiences helps organizations understand they can help shape people's experiences by starting with initial awareness (entice stage), the experience of use (enter and engage stages), and eventually becoming the stories and memories of the important events in life (exit and extend stages) (74). These frameworks, along with others (see Figure 5), account for real-life experiences that are often overlooked by formalized written policies, medical protocols, and scientific methods. Ignoring them can cause health initiatives like implementing new public health protocols, expanding the adoption of affordable policies, and increasing healthcare service access to fail to create sustainable relief or transformative change (75, 76).

Rigorously applied, design knowledge from HCD, strategic design, and other design approaches can help organizations change their structures and processes (77, 78). In the health industry, for example, the Mayo Clinic, Blue Cross Blue Shields Massachusetts, Walgreens, and other organizations are leveraging design beyond product development, including strategic planning, operational efficiency and quality, digitalization of internal processes, and patient and healthcare workers experiences. While designers have helped health-related organizations overcome specific challenges at the project level, literature reviews have recognized little evidence of design helping expand knowledge at the field level (79, 80).

The confluence of design and public health knowledge is still nascent, and the know-how and competencies that organizations urgently need are not readily available. Currently, a few initiatives are undertaking this mission, such as the Design for Health, funded by USAID and the Bill and Melinda Gates Foundation (81, 82) and the Design Laboratory, or D-Lab, at the Harvard T.H. Chan School of Public Health (83). While these and other efforts have the potential to help individuals and organizations envision new possibilities for disease governance, course, and burden previously not considered, there must be a critical and intentional investment in advancing anti-racist approaches to avoid transferring existing bias in both fields into future solutions.

Like public health, design is not free from anti-Black bias (84, 85). As seen in various disciplines, many in positions of power have been among the most privileged in society and thus often have less direct experience with the adverse effects of structural oppression and injustice that stratify society (86). This separation between positions of power and experience with oppression promotes the erasure of positionality, unconscious biases, and asymmetrical control of research inputs and outputs (87). Although recent efforts in design are intentionally exploring new directions that go against white norms and beliefs established by the lenses of white scholars, the journey is far from being completed (88, 89).

### Reoccurring Outcomes Requires Bold Interventions

We are in a transformational moment that requires solutions that are audacious in their optimism, speed, scale, and scope. What if we knew how to design anti-racist health systems? Which body of knowledge and database would we rely on for decision-making? What disciplines would shape these systems? What organizational models and leadership structures would inform health governance? What health protocols, guidelines, and practices would medical professionals use to accommodate and respond to the aspirations and health experiences of Black populations? How would these systems improve the health and well-being of Black people?

The increasing recognition of the various inequities conditioned by anti-Black racism suggests that public health will likely increase its impact if the field is able to work with other disciplines and domains of practice, including design. The questions posed in this work exemplify meaningful areas for future inquiry at the intersection of these two fields that challenge existing beliefs in health-related organizations and can help expand public health knowledge at the field level while guiding action at the project level. Their answers, however, require substantive historical, theoretical, and empirical work that goes beyond the scale and scope of this article. Furthermore, designing and implementing anti-racist systems is not a responsibility of only public health and design. Instead, it must be a criterion for revising and advancing the body of work from diverse fields influencing and conditioning the experiences of Black populations in the US and beyond.

Still, the urgent need for anti-racist health systems must not wait for answers from standard processes of knowledge creation or organizational change, often shaped by disciplinary silos in research and practice. On the contrary, the scope and scale of these problems require new flexible infrastructures that enable a growing network of diverse game-changers to work together and accelerate our way into more sustainable, transformative solutions.

This paper presented a new conceptual model that can help us all progress in this direction. The resulting model relates disease governance, course, and burden from public health with a design framing of organizational strategies, operations, offerings, and people's experiences. For each one of these relationships, we identified patterns of racist manifestations and used historical and contemporary examples to demonstrate how together they lead to inequitable health outcomes for Black populations in the US.

The patterns concerning disease governance involve strategic white framing of value creation opportunities, racial erasure in internal operations to realize value, and the consequent racial bias transferred into systems of offerings that shape health outcomes. There are two patterns highlighted in disease courses. One relates to bias in data captured and used by health-related organizations due to under-reporting and lack of documentation of disease endured by Black individuals. The other reflects a shift in responsibility that considers Black individuals accountable for dealing with a history of gross misconduct and miscarriage of justice still vivid in public health responses that carry racist affordances providing inferior care to Black people. Lastly, we presented three patterns concerning how Black populations experience disease burden. Rather than institutionalizing these experiences, these patterns are presented from the perspective of individuals that can't be, protect, and/or support themselves due to a conflation of public health and public safety, frontline worker status, and economic marginalization, respectively. Such a holistic approach can help public health embrace systemic complexity and people-centered perspectives pragmatically to deal with structural issues underlying anti-Black racism and improve health outcomes for all.

## Author Contributions

AE and AN developed the conception and design of this work. AE conducted a literature review and data interpretation. AN localized design methodology to support the approach. All authors leveraged collective critical revision and final approval of the work to be published.

## Conflict of Interest

The authors declare that the research was conducted in the absence of any commercial or financial relationships that could be construed as a potential conflict of interest.

## Publisher's Note

All claims expressed in this article are solely those of the authors and do not necessarily represent those of their affiliated organizations, or those of the publisher, the editors and the reviewers. Any product that may be evaluated in this article, or claim that may be made by its manufacturer, is not guaranteed or endorsed by the publisher.
